# TATA box binding protein and ribosomal protein 4 are suitable reference genes for normalization during quantitative polymerase chain reaction study in bovine mesenchymal stem cells

**DOI:** 10.5713/ajas.20.0238

**Published:** 2020-07-28

**Authors:** Si-Jung Jang, Ryoung-Hoon Jeon, Hwan-Deuk Kim, Jong-Chan Hwang, Hyeon-Jeong Lee, Seul-Gi Bae, Sung-Lim Lee, Gyu-Jin Rho, Seung-Joon Kim, Won-Jae Lee

**Affiliations:** 1 Department of Veterinary Theriogenology and Biotechnology, College of Veterinary Medicine, Gyeongsang National University, Jinju 52828, Korea; 2 Department of Veterinary Theriogenology, College of Veterinary Medicine, Kyungpook National University, Daegu 41566, Korea; 3 Department of Veterinary Research, Daegu Metropolitan City Institute of Health & Environment, Daegu 42183, Korea; 4 Department of Veterinary Internal Medicine, College of Veterinary Medicine, Kyungpook National University, Daegu 41566, Korea

**Keywords:** Bovine, Mesenchymal Stem Cells, Reference Gene, Normalization, Quantitative Polymerase Chain Reaction

## Abstract

**Objective:**

Quantitative polymerase chain reaction (qPCR) has been extensively used in the field of mesenchymal stem cell (MSC) research to elucidate their characteristics and clinical potential by normalization of target genes against reference genes (RGs), which are believed to be stably expressed irrespective of various experimental conditions. However, the expression of RGs is also variable depending on the experimental conditions, which may lead to false or contradictory conclusions upon normalization. Due to the current lack of information for a clear list of stable RGs in bovine MSCs, we conducted this study to identify suitable RGs in bovine MSCs.

**Methods:**

The cycle threshold values of ten traditionally used RGs (18S ribosomal RNA [*18S*], beta-2-microglobulin [*B2M*], H2A histone family, member Z [*H2A*], peptidylprolyl isomerase A [*PPIA*], ribosomal protein 4 [*RPL4*], succinate dehydrogenase complex, subunit A [*SDHA*], beta actin [*ACTB*], glyceraldehyde-3-phosphate dehydrogenase [*GAPDH*], TATA box binding protein [*TBP*], and hypoxanthine phosphoribosyltrasnfrase1 [*HPRT1*]) in bovine bone marrow-derived MSCs (bBMMSCs) were validated for their stabilities using three types of RG evaluation algorithms (geNorm, Normfinder, and Bestkeeper). The effect of validated RGs was then verified by normalization of lineage-specific genes (fatty acid binding protein 4 [*FABP4*] and osteonectin [*ON*]) expressions during differentiations of bBMMSCs or POU class 5 homeobox 1 (*OCT4*) expression between bBMMSCs and dermal skins.

**Results:**

Based on the results obtained for the three most stable RGs from geNorm (*TBP*, *RPL4*, and *H2A*), Normfinder (*TBP*, *RPL4*, and *SDHA*), and Bestkeeper (*TBP*, *RPL4*, and *SDHA*), it was comprehensively determined that *TBP* and *RPL4* were the most stable RGs in bBMMSCs. However, traditional RGs were suggested to be the least stable (*18S*) or moderately stable (*GAPDH* and *ACTB*) in bBMMSCs. Normalization of *FABP4* or *ON* against *TBP*, *RPL4*, and *18S* presented significant differences during differentiation of bBMMSCs. However, although significantly low expression of *OCT4* was detected in dermal skins compared to that in bBMMSCs when *TBP* and *RPL4* were used in normalization, normalization against *18S* exhibited no significance.

**Conclusion:**

This study proposes that *TBP* and *RPL4* were suitable as stable RGs for qPCR study in bovine MSCs.

## INTRODUCTION

Mesenchymal stem cells (MSCs) have received attention in the fields of cell-based regenerative medicine and biotechnology, which is due to their advantages such as the need for ethical issues, easy accessibility, self-renewal property, multi-differentiation potentials, and immunomodulatory capacity [1,2]. Like numerous studies on MSCs in various species, bovine MSCs have also been widely investigated for the past few decades to elucidate their characteristics and verify their clinical potential [3–5]. In particular, as large animal models, including cattle, possess a greater similarity to humans than small animals such as rodents, there has been an increase in the number of studies on large animals to more reliably understand the potential of the clinical application of MSCs [3].

Gene expression studies are indispensable in the field of cellular biology research as they enable researchers to identify the gene regulatory network in cells [6]. In this context, quantitative real-time polymerase chain reaction (qPCR), which has the advantages of convenience, sensitivity, reproducibility, and reliability, has been most commonly used to verify the potential of MSCs and determine the change in the mRNA expression of genes of interest (GOIs) [1,2]. During qPCR, the GOI is normalized against a reference gene (RG), also known as a housekeeping gene, as an internal control for its relative quantification; this step corrects sample-to-sample variations in the context of different experimental conditions, sample quality, operators, and laboratories [1,2,7,8]. Therefore, RGs should be stably expressed in various samples and not be affected by various experimental conditions; in principle, RGs play a pivotal role in the vital functions of cell survival and maintenance [7,9].

However, till date, no single RG has been addressed to be universal and perfectly constant. It is known that the expression of RGs is also variable depending on the experimental conditions and cell types [10]. In particular, the normalization of GOIs against inadequate or unstable RGs may result in false or contradictory conclusions [1,7,11]. Therefore, validation of RGs for their stability under each experimental condition is an extremely important and prerequisite step for obtaining reliable results during qPCR assay [2,7,8].

Unfortunately, information for a clear list of stable RGs in bovine MSCs is currently lacking, despite the fact that studies on cattle are being widely conducted. Therefore, the primary objective of this study was to identify the most suitable RGs in bovine MSCs, before conducting further gene expression study by qPCR. After establishing bovine bone marrow-derived MSC lines, we evaluated the stability of a set of ten traditionally used RGs (18S ribosomal RNA [*18S*], beta-2-microglobulin [*B2M*], H2A histone family, member Z [*H2A*], peptidylprolyl isomerase A [*PPIA*], ribosomal protein 4 [*RPL4*], succinate dehydrogenase complex, subunit A [*SDHA*], beta actin [*ACTB*], glyceraldehyde-3-phosphate dehydrogenase [*GAPDH*], TATA box binding protein [*TBP*], and hypoxanthine phosphoribosyltrasnfrase1 [*HPRT1*]) using the three most well-known algorithms (geNorm, Normfinder, and Bestkeeper). Thereafter, we applied the most and least stable RGs by normalization of GOIs (fatty acid binding protein 4 [*FABP4*], osteonectin [*ON*], and POU class 5 homeobox 1 (*OCT4*) to verify the importance of selecting suitable RGs in each study.

## MATERIALS AND METHODS

### Ethics statement

All experimental procedures were approved by the Institutional Animal Care Use Committee at Kyungpook National University (approval number: 2020-0038).

### Chemicals and media

All chemicals and media were purchased from Thermo Fisher Scientific (Waltham, MA, USA), unless otherwise specified.

### Sample preparation

The bone marrow extracts and dermal skins from the femurs were obtained from ~3-year-old castrated male bulls (Hanwoo, bos taurus coreanae, n = 4) at the local abattoir after slaughtering. Samples from healthy individuals were collected only under veterinarian examination. The bovine bone marrow-derived MSCs (bBMMSCs, n = 4) were isolated and established according to previous reports [1]. In brief, the bone marrow extracts were aspirated using a bone marrow aspiration needle (Jamshidi, BD, Franklin Lakes, NJ, USA) with flushing by Dulbecco’s phosphate-buffered saline and centrifugated by the Ficoll (Ficoll Paque PLUS, GE Health care, Uppsala, Sweden) gradient method at 400 *g* for 30 min at 4°C. Then, the mononuclear cell fraction was harvested and plated onto culture flasks. Once the adherent cells on the culture flasks were observed, the supernatant was removed and changed with fresh culture media. The cells were cultured in advanced Dulbecco’s modified Eagle medium (ADMEM) containing 10% fetal bovine serum (FBS), 1% GlutaMax, 10 ng/mL basic fibroblast growth factor, and 1% penicillin–streptomycin (Pen-Strep) at 38.5°C in a humidified incubator at 5% CO_2_ in air. When ~80% confluence was reached, the cells were subcultured until passage 3 for further analysis. Small pieces of dermal skin (1 cm×1 cm, n = 4) at the femurs were collected, immediately preserved by snap-freezing with liquid nitrogen, and stored in a deep freezer until further experiment.

### Characterization of bBMMSCs

The morphological characteristics of the ~80% confluent cells at passage 3 were examined to assess whether they exhibited fibroblastic morphologies with dendritic spindle shapes. Then, the cells were harvested, fixed with 4% paraformaldehyde (PFA) at 4°C overnight, and incubated with fluorescein isothiocyanate (FITC)-conjugated mouse anti-bovine CD44 (1:10 dilution) and mouse anti-bovine CD45 (1:10 dilution) antibodies at room temperature for 1 h. A total number of 1×10^4^ FITC-labeled cells (%) was counted by flow cytometry (BD FACS Calibur, BD, USA). As mentioned in a previous report, the cells at passage 3 were differentiated for 3 weeks toward adipocytes or osteoblasts in an adipogenic medium (DMEM supplemented with 10% FBS, 100 mM indomethacin, 10 mM insulin, and 1 mM dexamethasone) or an osteogenic medium (DMEM supplemented with 10% FBS, 200 mM ascorbic acid, 10 mM glycerophosphate, and 0.1 mM dexamethasone), respectively [12]. The differentiated bBMMSCS were fixed with 4% PFA and stained with 0.5% oil red solution or 5% silver nitrate solution (Von Kossa staining) with 0.5% alizarin red solution to assess adipogenesis or osteogenesis, respectively.

### RNA extraction and cDNA synthesis

The qPCR-related procedures were conducted according to previous reports [1,12]. Total RNA was extracted from bBMMSCs, differentiated bBMMSCs toward adipocytes and osteoblasts, and deep-frozen dermal skins using a QIA shredder column and RNeasy mini Kit (Qiagen, Hilden, Germany), including the RNase-free DNase treatment step for 15 min to remove residual genomic DNA, in accordance with the manufacturer’s instructions. The concentration and purity of total RNA samples were quantified by assessing the A260/A280 ratio using a spectrophotometer (NanoDrop 1000), and only pure total RNA samples within 2±0.2 ratio were selected. First-strand cDNA was synthesized using 1 μg total RNA, 4 units Omniscript Reverse Transcriptase (Qiagen, Germany), 10 units RNase inhibitor, and 1 mM oligo dT primer at 60°C for 1 h using a thermal cycler (Qiagen, Germany).

### Primer efficiency and cycle threshold value acquisition

Considering the most common RGs in MSCs from other species, ten types of RGs were selected and designed using the NCBI Primer Designing Tool (http://www.ncbi.nlm.-nih.gov/tools/primer-blast/), resulting in a PCR amplicon with 80 to 130 base pairs at an annealing temperature of 60°C (Table 1) [1,7–9]. The qPCR was conducted using a Rotor Gene Q qPCR machine (Qiagen, Germany) with Rotor-Gene 2× SYBR Green mix (Qiagen, Germany), including 0.1 μg cDNA per reaction and 0.5 mM forward and reverse primers of RGs. The qPCR program designed to obtain the cycle threshold (Ct) values for each RG in bBMMSCs consisted of predenaturation at 95°C for 10 min; 45 PCR cycles at 95°C for 10 s, 60°C for 6 s, and 72°C for 4 s; melting curve from 60°C to 95°C at 1°C/s; and cooling at 40°C for 30 s. Amplification curves, melting curves, and Ct values were analyzed using the Rotor Gene Q Series Software (Qiagen, Germany). In addition, the size and specificity of all amplicons were checked by electrophoresis using 1% agarose gel with 0.1 mg/mL ethidium bromide. These qPCR experiments were repeated in triplicates. To validate the PCR efficiency of each RG in bBMMSCs, a standard curve of each primer of RG was generated from the Ct values using a four-fold serial dilution of cDNA from bBMMSCs under the aforementioned qPCR condition. The values related to PCR efficiency (E) and correlation (R^2^) were obtained using Excel (Microsoft, Redmond, WA, USA) as described in a previous report [7].

### Determination of stable RGs using geNorm, Normfinder, and Bestkeeper

The obtained Ct values of RGs in bBMMSCs from qPCR were analyzed for their stabilities using the three most well-known algorithms (geNorm, Normfinder, and Bestkeeper). The geNorm program calculates the stability measurement M (M value) for each RG. After a RG with the highest M value, indicating the least stable RG, is excluded from the pool of RGs, a new M value is continuously recalculated using the pool of left out RGs until the last two RGs with the lowest M value remained, implying the most stable RGs. In addition, geNorm calculates the normalization factor (NF) for each RG and proposes the optimal number of RGs for normalization (optimal normalization factor, NF_opt_) by continuously calculating the pairwise variation (V_n/n+1_) between consecutively ranked NF (NF_n_ and NF_n+1_) [13]. Normfinder is based on an analysis of variance-based model to estimate intra- and inter-group variations to estimate the most stable RG. In this analysis, a lower value from the pool of RGs indicates a RG with a higher stability. Moreover, it can suggest the best combination of two RGs for the normalization step [14]. The Bestkeeper algorithm evaluates the standard deviation (SD) and the coefficient of variance of Ct values of RGs using Pearson’s pairwise coefficient correlations of all RGs against each other. In this program, a gene with a SD >1.0 is considered as an unacceptable RG, and a lower value of SD (±Ct) implies a more stable RG [15].

### Application of different reference genes to normalization

For the purpose of verifying the effect of stability of RGs, the most and least stable RGs in the present study were applied to the normalization of lineage-specific gene (*FABP4* for adipogenesis and *ON* for osteogenesis) expressions in bBMMSCs during differentiation, and *OCT4* expression as a pluripotent marker in bBMMSCs and dermal skins. The aforementioned qPCR conditions were applied to obtain the Ct value of RGs and GOIs (*FABP4*, *ON*, and *OCT4*). Thereafter, the Ct values of GOIs were normalized against those of several RGs. Details regarding the primer of GOIs are described in Table 1.

### Statistical analysis

Pearson’s correlation analysis between NF_opt_ and NF for the three most stable RGs (NF_3_) was conducted, and Student’s *t*-test was applied to assess the relative GOIs expression using PASW Statistics 18 (SPSS Inc., Chicago, IL, USA). Significant differences were considered at p<0.01.

## RESULTS

### Characterization of bBMMSCs

**Figure 1:**
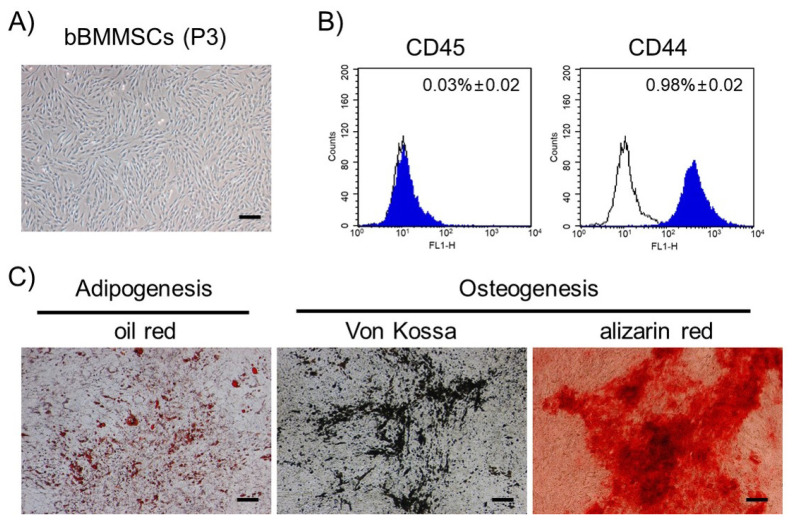
Characterization of bBMMSCs (magnification: ×40; bars: 100 μm). (A) The bBMMSCs at passage 3 (P3) exhibited fibroblastic morphologies with dendritic spindle shapes. (B) Positive expression of MSC-specific cell surface molecule (CD44) and absence of hematopoietic cell surface molecule (CD45) were identified in bBMMSCs by flow cytometry. The ratios were presented as mean%±standard error of the mean. (C) The bBMMSCs demonstrated differentiation potentials toward adipocytes (oil red staining) and osteoblasts (Von Kossa and alizarin red staining). bBMMSCs, bovine bone marrow-derived mesenchymal stem cells.

Figure 1 shows the results of characterization of bBMMSCs. The MSCs proliferated as adherent cells in the culture flasks and exhibited fibroblastic morphologies with dendritic spindle shapes (Figure 1A). CD44, a MSC-specific surface molecule, was strongly positive, whereas CD45, a hematopoietic stem cell marker, was negatively expressed in bBMMSCs (Figure 1B). The differentiation potentials into adipocytes or osteoblasts were determined, which showed the formation of lipid droplets or deposition of minerals, respectively (Figure 1C). Therefore, the homogenous population was confirmed to be pluripotent bBMMSCs and used in the present study.

### Examination of primer specificity, amplicon size, and primer efficiency

**Figure 2:**
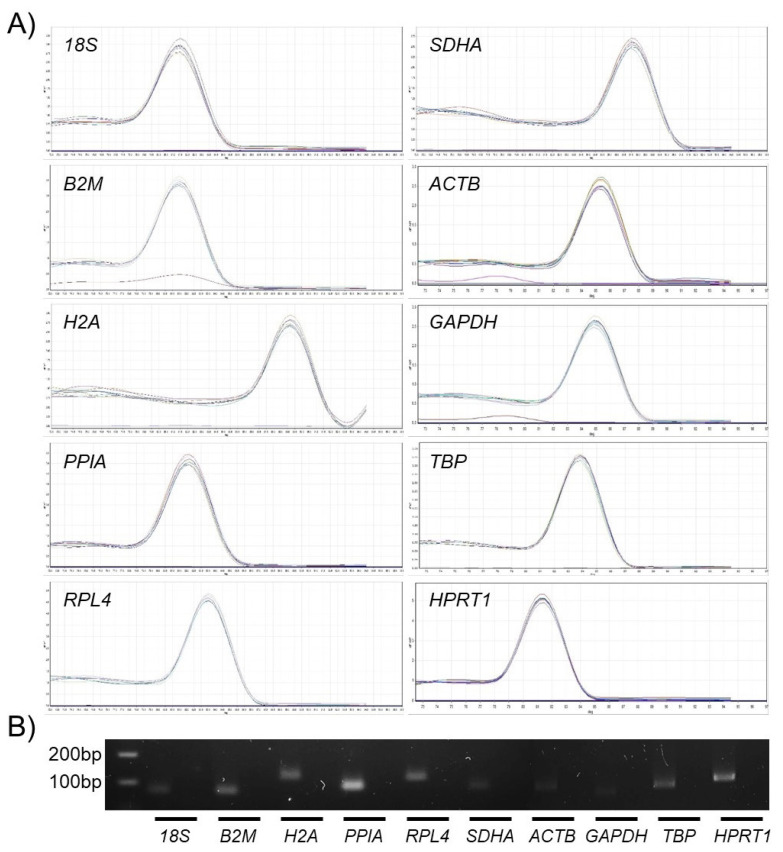
Examination of primer specificity and amplicon size. (A) A high peak of single products without any nonspecific amplification was identified during melting curve analysis. (B) Expected product size without nonspecific amplification was confirmed by gel electrophoresis. Lanes show ladder (100 and 200 bp) and respective amplicons (left) and negative controls (right) from each reference gene (RG).

In the melting curve analysis conducted to validate the primer specificity after qPCR, all reactions confirmed a high peak of single products without any nonspecific amplification (Figure 2A). In addition, the gel electrophoresis of the amplicons demonstrated an expected product size without nonspecific amplification such as primer dimers and multiple bands (Figure 2B, Table 1). The standard curve derived from the Ct values using a four-fold serial dilution of cDNA from bBMMSCs produced correlations (R^2^) of 0.991 to 0.998 and PCR efficiencies (E) of 0.95 to 1.04, implying that the primer design of the ten RGs in the present study was acceptable for qPCR. The detailed information of Ct values, correlation (R^2^), and PCR efficiencies (E) of each RG is described in Table 2.

### Analysis of stability of reference genes by geNorm, Normfinder, and Bestkeeper

**Figure 3:**
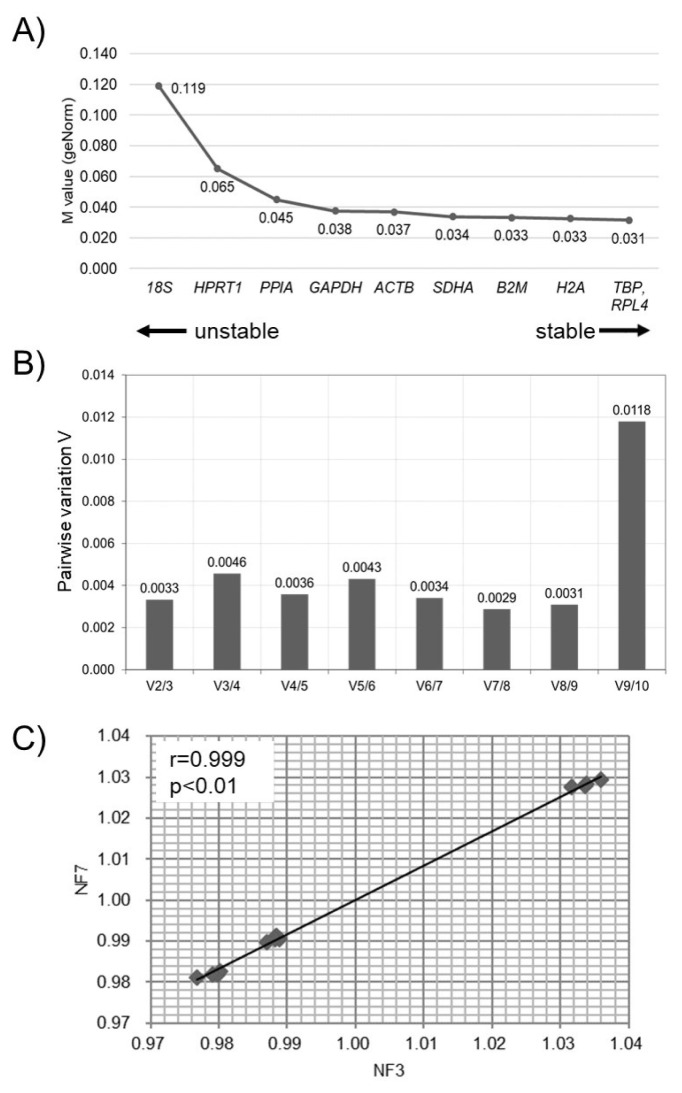
Analysis of stable RGs by geNorm. (A) The ranking of stability (M values) of RGs in bBMMSCs is presented from the least stable RG (left side of the graph) to the most stable RGs (right side of the graph). (B) The optimal number of RGs (NF_opt_) during normalization in bBMMSCs was recommended as 7 RGs (NF_7_) by pairwise variation (V_7/8_). (C) High correlation (*r* = 0.999, p<0.01) between NF_3_ and NF_7_ was identified by Pearson’s correlation analysis. RGs, reference genes; bBMMSCs, bovine bone marrow-derived mesenchymal stem cells; NF, normalization factor; V_n/n+1_, pairwise variation between consecutively ranked NF (NF_n_ and NF_n+1_); NF_opt_, NF for optimal number of RGs; NF_7_, NF_opt_ as 7 RGs; NF_3_, NF for the three most stable RGs.

**Figure 4:**
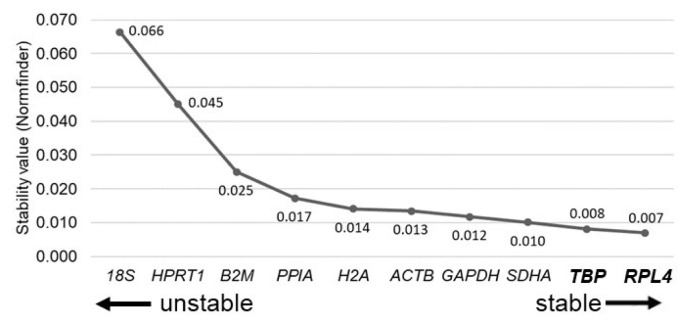
Identification of stable RG by Normfinder. The stability of RGs is ranked from the least stable RG (left side of the graph) to the most stable RG (right side of the graph). The most stable combination of RGs is presented in bold letters. RGs, reference genes.

**Figure 5:**
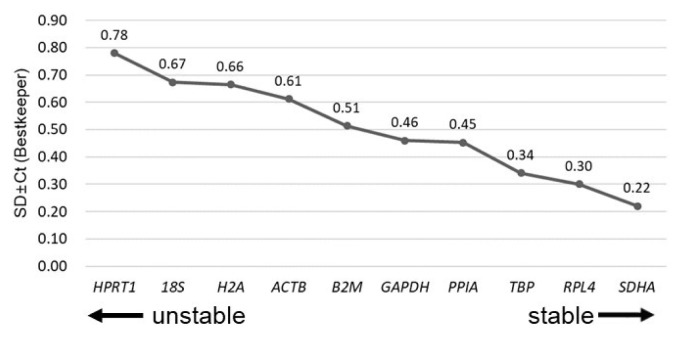
Verification of stable RGs by Bestkeeper. The ranking of stability (SD±Ct) of RGs from the least stable RG (left side of the graph) to the most stable RG (right side of the graph) was assessed. RGs, reference genes; Ct, cycle threshold; SD±Ct, standard deviation of the Ct.

The Ct values of the ten RGs in bBMMSCs were assessed for stability (M values) and NF_opt_ by geNorm (Figure 3). *TBP*, *RPL4*, and *H2A* were identified as the three most stable RGs in bBMMSCs, whereas the traditionally used RGs were determined as the least stable (*18S*) or moderately stable (*GAPDH* and *ACTB*) (Figure 3A). Furthermore, pairwise variation (V_n/n+1_) suggested that the set of seven RGs (V_7/8_) was considered as NF_opt_ (Figure 3B). As it was highly excessive to use the seven RGs in a normalization step in qPCR, we further analyzed the correlation of NF between NF_3_ and NF_opt_ (NF_7_) to reduce inefficient usage of RGs. Pearson’s correlation analysis revealed a high correlation (*r* = 0.999, p<0.01) between NF_3_ and NF_opt_, indicating that the three most stable RGs (*TBP*, *RPL4*, and *H2A*) were sufficient for normalization during qPCR procedures in bBMMSCs (Figure 3C). Similar to the results of geNorm, *TBP*, *RPL4*, and *SDHA* were determined as the three most stable RGs, and the least stable RG was *18S* in bBMMSCs according to Normfinder (Figure 4). In addition, the Normfinder algorithm suggested that *TBP* and *RPL4* comprised the most stable combination of two RGs for normalization. There were no RGs with a SD >1.0 in the Bestkeeper analysis, indicating the credibility of the RG candidates in the present study. Bestkeeper showed that the three most stable RGs with the three lowest SD (±Ct) values were *SDHA*, *RPL4*, and *TBP*. *18S* was also one of the least stable RGs, and *GAPDH* and *ACTB* were determined as moderately stable RGs in bBMMSCs (Figure 5). Altogether, based on the results obtained from geNorm, Normfinder, and Bestkeeper, it can be comprehensively concluded that *TBP* and *RPL4* were the two most stable RGs and the traditional RGs were the least stable (*18S*) or moderately stable (*GAPDH* and *ACTB*) in bBMMSCs. The small discrepancies, ranking of stability, from each program may have been possibly caused due to the use of different algorithms.

### Application of different reference genes to normalization

**Figure 6:**
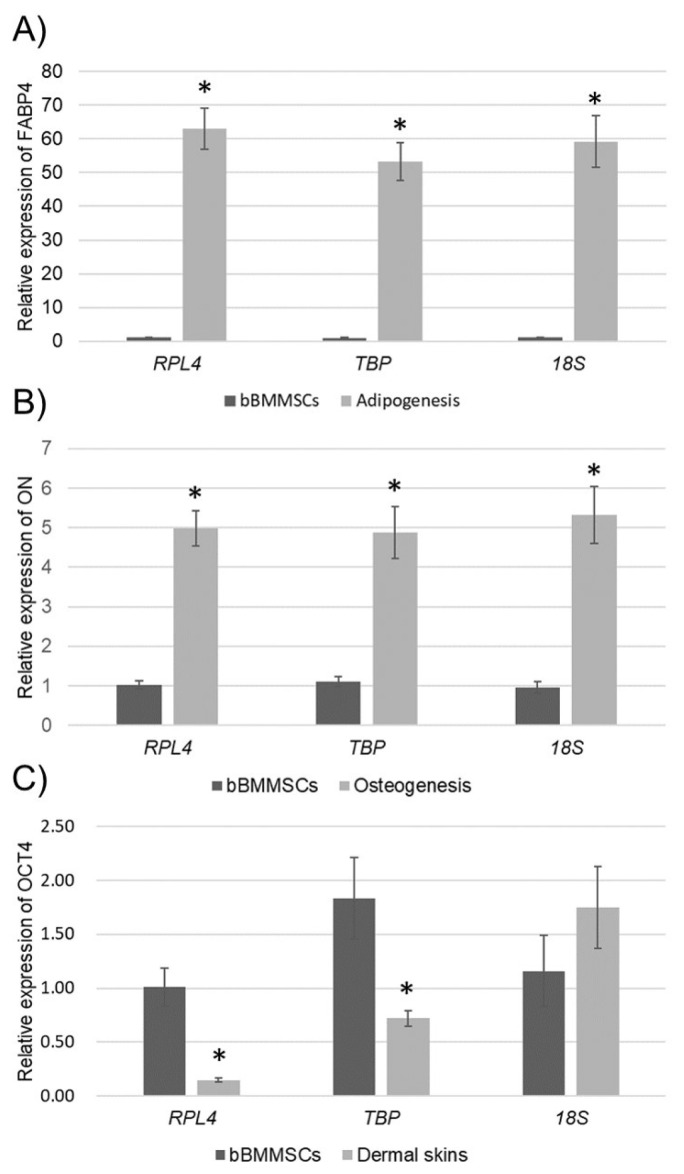
Application of different RGs to normalization. (A and B) Relative expression levels of lineage-specific genes (*FABP4* for adipogenesis and *ON* for osteogenesis) were normalized against the most stable RGs (*TBP* and *RPL4*) and the least stable RG (*18S*) in bBMMSCs during differentiation. (C) Relative expression level of *OCT4* was normalized against *TBP*, *RPL4*, and *18S* in bBMMSCs and dermal skins to verify the effect of stability of RGs. RGs, reference genes; *FABP4*, fatty acid binding protein 4; *ON*, osteonectin; *TBP*, TATA box binding protein; *RPL4*, ribosomal protein 4; *OCT4*, POU class 5 homeobox 1; bBMMSCs, bovine bone marrow-derived mesenchymal stem cells. Significant (p<0.01) differences between bBMMSCs and their counterparts are presented with asterisk.

When suitable (*TBP* and *RPL4*) and unsuitable (*18S*) RGs were selected using the three programs, they were used for the normalization of lineage-specific gene (*FABP4* and *ON*) expressions in bBMMSCs during differentiation or *OCT4* expression in bBMMSCs and dermal skins to confirm the effect of stability of RGs (Figure 6). It has been well known that differentiation-induced MSCs highly express the relevant lineage-specific markers such as *FABP4* and *ON*. Furthermore, because the dermal skins were considered as a completely differentiated tissues, we believed that the expression of the pluripotent marker (*OCT4*) could be lower or absent in the dermal skins compared to that in MSCs. As expected, when *TBP*, *RPL4*, and *18S* were employed for normalization, the expression of lineage-specific genes was significantly increased after differentiation inductions of bBMMSCs (Figure 6A, 6B). In addition, a significantly (p<0.01) lower expression of *OCT4* was detected in the dermal skins than in bBMMSCs when *TBP* and *RPL4* were used for normalization. In contrast, the normalization of *OCT4* against *18S* exhibited no significance (Figure 6C). These findings implied that invalidated RG could occasionally produce the unexpected results, which were derived from unreliable normalization data.

## DISCUSSION

Although qPCR has been widely used to elucidate the characteristics of bovine MSCs and confirm their clinical potential, information for a clear list of stable RGs in bovine MSCs is currently not available. In the present study, we established bBMMSCs and investigated their Ct values using ten commonly used RGs. The Ct values were then assessed for stability using the three most well-known algorithms (geNorm, Normfinder, and Bestkeeper). Consequently, *TBP* and *RPL4* were found to be the two most stable RGs in bBMMSCs, but traditional RGs such as *18S*, *GAPDH*, and *ACTB* were determined to be less stable. These attempts to validate the suitable RGs in each experimental condition represent a prerequisite for the reliable assessment of gene expression by qPCR, as there is no equally and constantly expressed RG regardless of various experimental conditions and the usage of an inappropriate RG may lead to false or contradictory results [7,8,11]. In this respect, to the best of our knowledge, the present study is the first to validate stable RGs in bovine MSCs.

Using other bovine specimens, several studies have been conducted to validate the stability of RGs in each experimental condition. Consistent with the present study results depicted in Figure 3 to 5, *TBP* was stably expressed in several bovine tissues, including the cumulus cell [16], corpus luteum obtained from cyclic or pregnant cows [17], and liver and thyroid [18]. *RPL15*, a ribosomal protein family along with *RPL4*, was also found to show stable expression in oocytes collected from cattle during winter and summer [19]. In agreement with the results of the present study, the usage of *18S* for normalization was not recommended in the bovine muscular tissue [20] and corpus luteum [17]. In addition, several experimental conditions with cattle specimens in terms of the mammary gland under different lactation periods [21], polymorphonuclear leukocytes [22], cumulus cell [16], muscular tissue [20], and peripheral lymphocytes [23] were found to be unsuitable to use *ACTB* and *GAPDH* for the normalization step. On the other hand, using some conditions such as polymorphonuclear leukocytes [22] and embryos produced *in vitro* [24,25] in cattle, *18S* and *GAPDH* were validated as stable RGs, respectively. Altogether, the discrepancies in different stabilities of RGs in each report using cattle are believed to be caused due to the differences in experimental conditions. Therefore, validation of the stability of RGs under each experimental condition is considered as an essential step before analyzing bovine gene expression by qPCR [2,7,8].

Similar studies have also been conducted in MSCs from other species. Comprehensively, *TBP* was one among the three most stable RG in human [2] and porcine [1] MSCs regardless of cell source and differentiation induction, but *18S* was the least stable RG. While *RPL13A*, a ribosomal protein family along with *RPL4*, was found to be stable in human MSCs derived from adipose tissue [8], bone marrow, and fetal tissue [9], normalization with *ACTB* was not recommended due to instability.

A survey based on NCBI-PubMed data for the usage of traditional RGs reported that *GAPDH* (27.24%), *ACTB* (30.62%), and *18S* (12.52%) were the three most widely used RGs in qPCR, semi-qPCR, and northern blotting [26]. *GAPDH* is ubiquitously expressed in the cell and involved in DNA repair, tRNA export, membrane fusion, and transport, cytoskeletal dynamics, cell death, oligomerization, posttranslational modification, and subcellular localization [27]. *ACTB* is an indispensable component of the cytoskeleton in the cell for cell migration, cell division, and regulation of gene expression [28]. *18S* is a component of the ribosomal RNA and plays a role in the biogenesis and function of ribosome in the cell [29]. However, its expression level is dependent on the experimental condition, in spite of its vital functions of cell survival and maintenance. In detail, the Ct values of both *GAPDH* and *ACTB* were decreased in *in vitro* cultured blood mononuclear cells even without any treatment [30] and altered under differentiation induction [1] and long-term culture [7] in MSCs. In the present study, we demonstrated the effect of the validated RGs during normalization and highlighted the possibility of false or misleading result caused by the usage of traditional RGs without validation (Figure 6). Normalization with both the most (*TBP* and *RPL4*) and least (*18S*) RGs could generate significant increase of lineage-specific genes in the differentiated bBMMSCs. However, there was no significant difference in *OCT4* expression, which is known to be highly expressed in MSCs than in differentiated cells as a pluripotent marker, between bBMMSCs and dermal skins when the unstable RG (*18S*) was used for normalization. Similarly, although significant gradual downregulation of *OCT4* expression during long-term culture of human MSCs, indicating progressive reduction of pluripotency, after normalization against the most stable RGs was observed, the least stable RG (*GAPDH*) generated no difference in *OCT4* expression [7]. These findings indicated the importance of validation of RGs before using even though they are widely used RGs.

An ideal RG should be neither affected nor regulated by each experimental condition. However, as no single RG has till date been addressed to be universal and perfectly constant regardless of the experimental condition, the importance of validation of RGs before normalization cannot be emphasized enough to avoid generation of false or contradictory conclusions. To summarize, the present study proposes that *TBP* and *RPL4* were suitable as stable RGs for gene expression study in bovine MSCs. These results may contribute to the experimental set-up of researchers working on animal MSCs as reference data.

## Figures and Tables

**Table 1:** Information of primers used in the present study

Gene name (symbol)	Primer sequences	Product (bp)	Reference
18S ribosomal RNA (*18S*)	F: cgcggaaggatttaaagtg	89	XR_003508809.1
	R: aaacggctaccacatccaag		
Beta-2-microglobulin (*B2M*)	F: tccgccccagattgaaattg	81	NM_173893.3
	R: tccttgctgaaagacaggtctg		
H2A histone family, member Z (*H2A*)	F: ggtaaggctgggaaggactc	124	BC109743.1
	R: catggctggtcgtcctagat		
Peptidylprolyl isomerase A (*PPIA*)	F: aaaacttccgtgctctgagc	112	BC105173.1
	R: ttatggcgtgtgaagtcacc		
Ribosomal protein 4 (*RPL4*)	F: caagagtaactacaaccttc	122	XM_027553034.1
	R: gaactctacgatgaatcttc		
Succinate dehydrogenase complex, subunit A (*SDHA*)	F: cacacgctttcctatgtcgatg	94	NM_174178.2
	R: tggcacagtcagcttcattc		
Beta actin (*ACTB*)	F: ctcttccagccttccttcct	101	AY141970.1
	R: tagaggtccttgcggatgtc		
Glyceraldehyde-3-phosphate dehydrogenase (*GAPDH*)	F: agttcaacggcacagtcaag	82	NM_001034034.2
	R: ggatctcgctcctggaagat		
TATA box binding protein (*TBP*)	F: cgtgcccgaaatgctgaata	108	NM_001075742.1
	R: gcacaccatcttcccagaac		
Hypoxanthine phosphoribosyltrasnfrase1 (*HPRT1*)	F: agcgtggtgattagcgatga	126	NM_001034035.2
	R: ccgttcggtcctgtccataa		
Fatty acid binding protein 4 (*FABP4*)	F: cactccagatgacaggaaagtc	135	NM_174314
	R: acacattccagcaccatctt		
Osteonectin (*ON*)	F: gagggcctggatcttctttc	101	NM_174464
	R: cggtttcttccaccacttct		
POU class 5 homeobox 1 (*OCT4*)	F: gtggaggaagctgacaacaa	87	NM_174580.3
	R: actcgtccgctttctctttc		

**Table 2:** Information of Ct values, correlation (R^2^) and polymerase chain reaction efficiencies (E) of each reference genes

Gene	Ct value (mean±SEM)	Correlation (R^2^)	PCR efficiencies (E)
*RPL4*	29.3±0.2	0.995	0.98
*H2A*	23.5±0.2	0.998	1.02
*PPIA*	20.5±0.1	0.995	0.98
*S18*	10.9±0.2	0.992	0.97
*B2M*	27.3±0.2	0.991	0.96
*SDHA*	31.2±0.1	0.997	1.04
*ACTB*	18.1±0.2	0.997	0.99
*GAPDH*	17.9±0.1	0.994	0.98
*TBP*	26.1±0.2	0.995	1.01
*HPRT1*	23.3±0.3	0.992	0.95

Ct, cycle threshold; PCR, polymerase chain reaction; SEM, standard error of the mean; *RPL4*, ribosomal protein 4; *H2A*, H2A histone family, member Z; *PPIA*, peptidylprolyl isomerase A; *S18*, 18S ribosomal RNA; *B2M*, beta-2-microglobulin; *SDHA*, succinate dehydrogenase complex, subunit A; *ACTB*, beta actin; *GAPDH*, glyceraldehyde-3-phosphate dehydrogenase; *TBP*, TATA box binding protein; *HPRT1*, hypoxanthine phosphoribosyltrasnfrase1.
